# Radiosensitization of Prostate Cancers *In Vitro* and *In Vivo* to Erbium-filtered Orthovoltage X-rays Using Actively Targeted Gold Nanoparticles

**DOI:** 10.1038/s41598-017-18304-y

**Published:** 2017-12-22

**Authors:** Allison M. Khoo, Sang Hyun Cho, Francisco J. Reynoso, Maureen Aliru, Kathryn Aziz, Monica Bodd, Xi Yang, Md F. Ahmed, Selcuk Yasar, Nivedh Manohar, Jongmin Cho, Ramesh Tailor, Howard D. Thames, Sunil Krishnan

**Affiliations:** 1 Department of Experimental Radiation Oncology, The University of Texas MD Anderson Cancer Center, Houston, Texas USA; 2 Department of Radiation Physics, The University of Texas MD Anderson Cancer Center, Houston, Texas USA; 3 Department of Imaging Physics, The University of Texas MD Anderson Cancer Center, Houston, Texas USA; 4 Department of Radiation Oncology, The University of Texas MD Anderson Cancer Center, Houston, Texas USA; 5 Department of Biostatistics, The University of Texas MD Anderson Cancer Center, Houston, Texas USA; 6 Present Address: A.M.K.: Baylor College of Medicine, Houston, Texas USA; 7 Present Address: F.J.R.: Dept. of Radiation Oncology, Washington University, St. Louis, Missouri USA; 8 Present Address: N.M.: Dept. of Radiation Oncology, Emory University, Atlanta, Georgia USA; 9 Present Address: J.C.: Dept. of Physics, Oklahoma State University, Still Water, Oklahoma USA

## Abstract

Theoretical investigations suggest that gold nanoparticle (GNP)-mediated radiation dose enhancement and radiosensitization can be maximized when photons interact with gold, predominantly via photoelectric absorption. This makes ytterbium (Yb)-169, which emits photons with an average energy of 93 keV (just above the *K-*edge of gold), an ideal radioisotope for such purposes. This investigation tests the feasibility of tumor-specific prostate brachytherapy achievable with Yb-169 and actively targeted GNPs, using an external beam surrogate of Yb-169 created from an exotic filter material - erbium (Er) and a standard copper-filtered 250 kVp beam. The current *in vitro* study shows that treatment of prostate cancer cells with goserelin-conjugated gold nanorods (gGNRs) promotes gonadotropin releasing hormone receptor-mediated internalization and enhances radiosensitivity to both Er-filtered and standard 250 kVp beams, 14 and 10%, respectively. While the degree of GNP-mediated radiosensitization as seen from the *in vitro* study may be considered moderate, the current *in vivo* study shows that gGNR treatment plus Er-filtered x-ray irradiation is considerably more effective than radiation treatment alone (p < 0.0005), resulting in a striking reduction in tumor volume (50% smaller) 2 months following treatment. Overall, the current results provide strong evidence for the feasibility of tumor-specific prostate brachytherapy with Yb-169 and gGNRs.

## Introduction

The goal of radiation therapy (RT) is to deliver cytotoxic doses of radiation to tumor targets while sparing surrounding normal tissue. Over the years, the ability to focus high dose radiation physically on tumors has gradually improved with technological advances in RT devices. On the other hand, a fundamentally different strategy to sensitize tumor cells to radiation via molecular targeting remains inadequately explored in the clinic. A variety of materials including chemotherapeutic agents, oxidant stressors, and nanomaterials continue to be investigated for their ability to specifically sensitize tumor cells to RT^1–3^. In particular, gold nanoparticles (GNPs), with sizes ranging from 1–100 nm, represent an attractive therapeutic option as they are biologically compatible, far smaller than the typical pore size found in leaky tumor vasculature^4,5^, and very effective in terms of inducing physical dose enhancement around themselves due to the high atomic number (Z) of gold (Z = 79)^6,7^. Indeed, multiple *in vivo* studies have demonstrated that GNPs, even when intravenously administered, preferentially accumulate within tumors due to the enhanced permeability and retention (EPR) effect and offer a varying degree of physical radiation dose enhancement to tumor targets^7–11^.

The degree and specificity of GNP-mediated dose enhancement depend largely on the amount and spatial distribution of GNPs that are present within tumors and internalized by tumor cells. In earlier attempts such as the first successful animal study by Hainfeld *et al*.^7^, passive targeting via the EPR effect was typically utilized to accumulate high concentrations of GNPs (on the order of 1 mg/g) within tumors, followed by immediate kilovoltage (e.g., 250 kVp) x-ray irradiation, in order to achieve clinically significant (>10%) *in vivo* radiosensitization. In subsequent attempts, GNPs are often coated with polyethylene glycol (“pegylated”) to decrease reticuloendothelial cell capture and conjugated to active cancer-specific targeting moieties to achieve favorable pharmacokinetics and durable localization to tumor cells^12^. Further experimentation using a variety of GNP shapes and sizes have shown differing rates of blood vessel extravasation and metabolic clearance^12,13^. Nanorods, for example, have been shown to offer superior intratumoral transport and distribution compared with nanospheres^14^.

GNP-mediated dose enhancement is especially relevant to the treatment of prostate cancer, one of the most common oncologic indications for radiation therapy. Furthermore, prostate cancer cells overexpress the human gonadotropin releasing hormone (GnRH) receptor, a molecular target exploited by goserelin, an FDA-approved GnRH receptor agonist. The commercial availability of a targeting moiety specific to prostate cancer has afforded an opportunity to examine actively-targeted GNPs in a relevant clinical context. To that end, recent work using gold nanorods (GNRs) conjugated to goserelin demonstrated that human prostate cancer cells internalize significantly more goserelin-conjugated pegylated GNRs (gGNR) than nonconjugated pegylated GNR (pGNR) as measured by both transmission electron microscopy (TEM) and inductively-coupled plasma mass spectrometry (ICP-MS)^8^. Furthermore, gGNR sensitized human prostate cancer cells to 6 MV x-ray radiation to a significantly higher degree than pGNR as measured by *in vitro* clonogenic survival and *in vivo* tumor growth measurements in a murine subcutaneous xenograft model^8^. Notably, these actively-targeted gGNRs mediated *in vivo* radiosensitization at a dose of 10 µg of gold per gram of body weight, over one hundred times less gold than the 1.7 mg/g dose used in initial GNP studies^7,12^.

When present in low-Z tissue-like media or water, GNPs interact with incident photons of the given energy at much higher probability for photoelectric absorption (proportional to ~ Z^3^) than the surrounding media. As a result, photoelectrons (PEs) and Auger electrons (AEs -including Coster-Kronig electrons) emitted from GNPs result in physical dose enhancement to the surrounding tissue-like media causing downstream DNA damage directly (double-strand breaks) or indirectly through ionization of intracellular water molecules into free radicals^15–20^. Photoelectric absorption is thought to be the predominant physical mechanism behind GNP-mediated dose enhancement and radiosensitization^6,12^ and the probability for photoelectric absorption interaction decreases rapidly with increasing photon energy (inversely proportional to ~E^3^). From a physical point of view, therefore, low energy photons in the keV range (e.g., kilovoltage x-rays) are preferred over high energy photons in the MeV range (e.g., 6 MV x-rays) in order to maximize the effects of GNP-mediated dose enhancement and radiosensitization^6,17,21,22^. Due to their short penetration depth in tissue-like media, however, kilovoltage x-ray beams appear to play a fairly limited role for clinical translations of GNP-mediated radiosensitization.

Meanwhile, it continues to remain a viable option to use various radioisotopes emitting photons (or gamma rays) predominantly in the keV range for further enhancing the probability of photoelectric absorption interactions with GNPs, while avoiding the aforementioned limitations of external kilovoltage x-ray beams by adopting brachytherapy approaches. In particular, previous Monte Carlo (MC) studies^17,23,24^ suggested ytterbium (Yb)-169 (half- life of 32.0 days) as an almost ideal radioisotope choice for brachytherapy implementation of GNP-mediated dose enhancement and radiosensitization. Specifically, Yb-169 has an intensity-weighted average gamma ray energy of about 93 keV, which is significantly lower than that (i.e., ~395 keV) of iridium (Ir)-192 (half-life of 73.8 days), a popular radioisotope choice for high dose rate brachytherapy, and close to the *K-*absorption edge of gold (i.e., ~80 keV). Consequently, Yb-169 gamma rays have a higher probability of producing *K-*shell PEs from GNPs presented within a tumor than Ir-192 gamma rays thereby resulting in larger GNP-mediated dose enhancement. Moreover, Yb-169 gamma rays can produce both gold *K-* and *L-*shell PEs, whereas much lower energy sources used in prostate brachytherapy such as palladium (Pd)-103 (half-life of 17.0 days; average energy of ~21 keV) and iodine (I)-125 (half-life of 59.4 days; average energy of ~28 keV) are capable of producing only *L-*shell PEs from GNPs. Many of these PEs due to Yb-169 gamma ray irradiation have longer ranges in tissue than those due to lower energy sources. As a result, Yb-169 source would result in more uniform GNP-mediated dose enhancement over a tumor volume and/or allow PEs generated from internalized GNPs to reach critical cellular organelles such as the nucleus more easily. Additionally, while the effectiveness of I-125 and Pd-103 for GNP-mediated dose enhancement and radiosensitization has also been shown in previous computational and preclinical studies^17,23,25,26^, low dose rate sources such as I-125 and Pd-103 used for permanent prostate implant would require a sustained release of GNPs or multiple injections of GNPs to warrant the level of GNP-mediated dose enhancement and radiosensitization as shown in the aforementioned studies, thus may not be considered practical for immediate clinical applications^17,21^.

Despite the current wealth of theoretical data supporting the effectiveness of Yb-169 for GNP-mediated dose enhancement and radiosensitization, however, there have been few observational studies evaluating the validity of such data so far. Thus, we have chosen prostate cancer for this validation, predicated on the frequent clinical use of low dose rate (I-125 and Pd-103) and high dose rate (Ir-192) brachytherapy in this disease and relative ease of improving an existing treatment modality rather than defining a new clinical paradigm altogether. Specifically, in this study, we aimed to test the feasibility of tumor-specific prostate brachytherapy achievable with Yb-169 and actively targeted GNPs. In particular, we investigated the role of the energy range of photons present in abundance within the gamma ray spectrum of Yb-169 for GNP-mediated radiosensitization through *in vitro* and *in vivo* studies. Due to the absence of a commercially available Yb-169 source providing sufficiently high dose rate necessary for *in vitro* and *in vivo* studies, we developed an external beam surrogate of Yb-169 using an exotic filter material – erbium (Er) and a standard copper (Cu)-filtered 250 kVp beam. We then used standard Cu-filtered 250 kVp beams with and without the Er-filter in our *in vitro* experiments to irradiate human prostate cancer cells pre-treated with GNPs, utilizing both actively targeted gGNRs and non-targeted pGNRs. Finally, we performed our *in vivo* experiments by irradiating mice bearing tumors (human prostate cancer xenograft) with a standard copper-filtered 250 kVp beam with the Er-filter. Overall, although not performed with an actual Yb-169 source, this investigation provided strong evidence for the feasibility of tumor-specific prostate brachytherapy with Yb-169 and gGNRs. Also, the current experimental approach and results helped deepen our understanding of the mechanism of GNP-mediated radiosensitization and will likely play a crucial role for translating the tumor-specific brachytherapy strategy to the clinic for prostate cancer as well as other types of cancers.

## Results

### Spectral Characteristics of Er-filtered 250 kVp X-ray Beam

**Figure 1:**
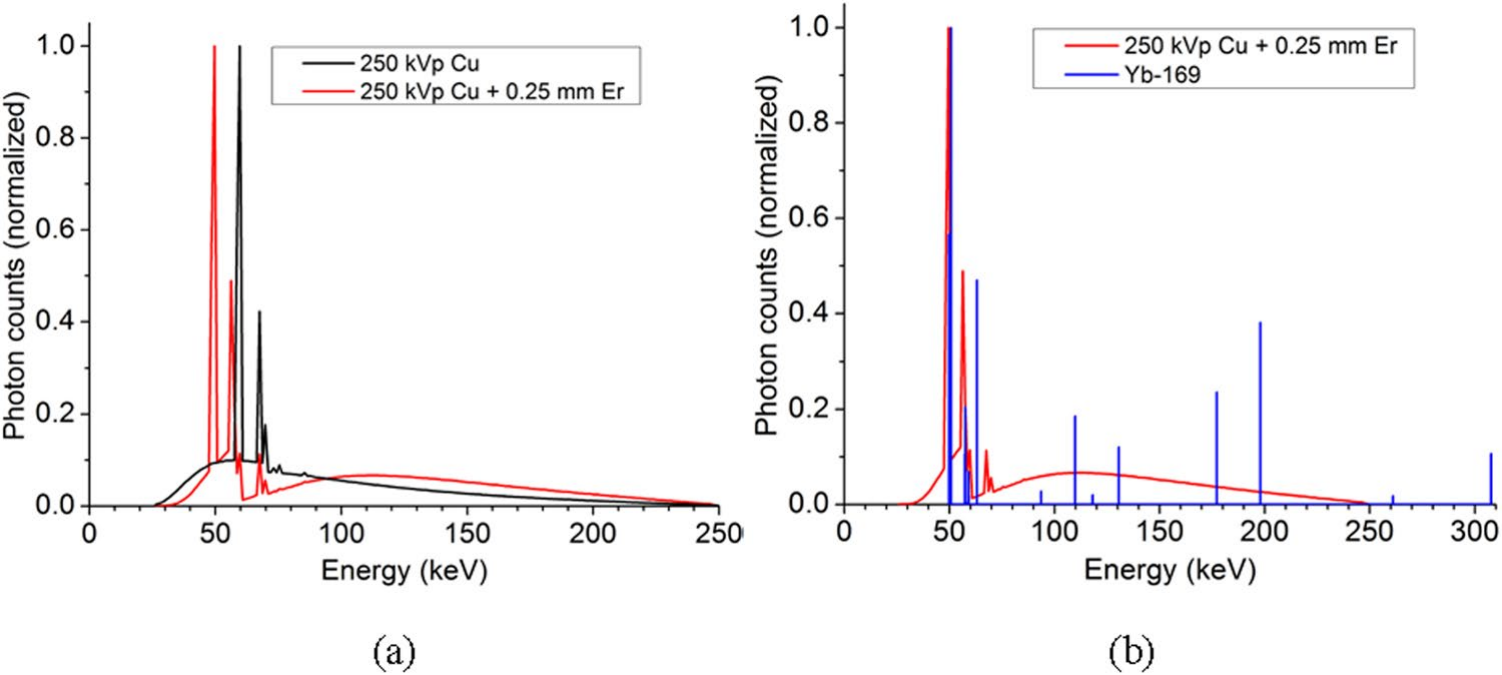
Comparison of photon spectra. (**a**) Photon spectrum of 250 kVp (Cu) compared to the same beam after the addition of a 0.25 mm erbium filter. The strong characteristic lines of tungsten on the original beam are suppressed and replaced with those of erbium at 49.1 keV and 55.7 keV. (**b**) Photon spectrum of 250 kVp (Cu) after the addition of a 0.25 mm erbium filter compared to the photon spectra of Yb-169. The fluorescence photons from erbium at 49.1 keV and 55.7 keV match the strongest part of the Yb-169 spectrum.

As shown in Fig. 1a, further filtration of a standard Cu-filtered 250 kVp beam using the 0.25 mm Er foil resulted in a replacement of the tungsten *K-*shell fluorescence (or characteristic) x-ray peaks with the erbium K-shell fluorescence (or characteristic) x-ray peaks. This spectral change allowed a better emulation of the Yb-169 gamma ray spectrum using the 250 kVp x-ray beam as an external beam surrogate, because the *K-*shell fluorescence x-ray peaks of erbium, *K*_*α*_ (49.1 keV) and *K*_*β*_ (55.7 keV), closely match the most intense spectral lines of the Yb-169 gamma ray spectrum (~49 and ~63 keV) (Fig. 1b). Note a perfect match between continuous bremsstrahlung x-ray and discrete gamma ray spectra cannot be produced by any practical means. Thus, the currently applied x-ray spectrum modulation strategy was deemed acceptable within the scope of this investigation.

### Secondary Electron Spectra Within a GNP-loaded Tumor

**Figure 2:**
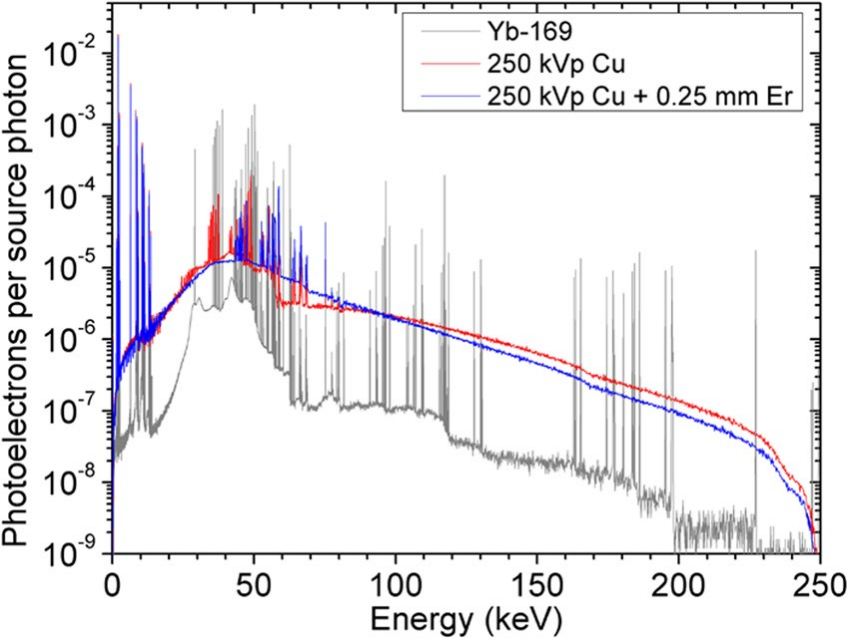
Spectra of photoelectrons released in the tumor region for each photon beam. Addition of an Er-filter to the standard (Cu-filtered) 250 kVp beam resulted in a sharp increase in the photoelectron yield around 50 keV within a tumor loaded with 7 mg Au/g tissue, thus providing a better match with the secondary electron spectrum associated with the Yb-169 gamma ray spectrum. Note the overall mismatch in the secondary electron spectra was due to the nature of two different types (continuous vs. discrete) of incident photon spectra.

The secondary electron spectra within a GNP-loaded tumor irradiated by various filtered 250 kVp x-ray beams as well as a hypothetical Yb-169 beam were calculated using the MC method (Fig. 2). The results are summarized in a more quantitative fashion in Table 1. Basically, despite the overall mismatch resulting from the nature of two different types (continuous vs. discrete) of incident photon spectra, the secondary electron spectrum associated with the Er-filtered 250 kVp x-ray beam showed a decreased photoelectron yield around ~57 keV due to the *K-*edge of erbium and a sharp increase in the photoelectron yield around 50 keV due to the *K-*shell fluorescence x-rays from erbium, improving the matching with the secondary electron spectrum associated with the Yb-169 gamma ray spectrum. The information presented in Table 1 gives insight into exactly how each beam locally deposits its energy and how close the correlation is between each one of the x-ray beams and the Yb-169 source. The results show the Yb-169 beam with the highest total energy deposition (sum of “Total PE Energy” and “Total AE Energy” in Table 1) of 1.08 keV per source photon. The Cu-filtered beam showed much closer agreement with 0.964 keV deposited per source photon, but still 11% lower than that of the Yb-169 source. On the other hand, the Er-filtered beam showed the best agreement with the Yb-169 with a total of 1.05 keV deposited per source photon and just 2.7% lower than the Yb-169 source. Note the MC results summarized in Table 1 serve mainly for a relative comparison among the considered photon spectra, in terms of GNP-mediated dose enhancement achievable under the given conditions (see the Method section) which were not identical to those present during the current *in vivo* experiment.

**Table 1:** Summary of MC simulations for the secondary electron spectra.

Radiation Source	Gold		Tissue		Total PE Energy (keV)	Gold	
	PE Yield	PE Energy (keV)	PE Yield	PE Energy (keV)		AE Yield	Total AE Energy (keV)
*Yb-169*	*0.0142*	*0.618*	*0.00930*	*0.365*	*0.983*	*0.0264*	*0.0983*
250 kVp *Cu*	*0.0139*	*0.564*	*0.00790*	*0.299*	*0.863*	*0.0257*	*0.101*
250 kVp *Cu* + *Er*	*0.0152*	*0.603*	*0.00964*	*0.334*	*0.937*	*0.0282*	*0.110*

Yield of photoelectrons (PE) and Auger/Coster-Kronig electrons (AE) and intensity-weighted total energy from gold and tissue for a tumor loaded with 7 mg Au/g of tissue for each radiation source. Auger/Coster-Kronig electrons are only produced in gold. All values listed in the table are normalized per source photon.

### pGNR and gGNR Characterization

**Figure 3:**
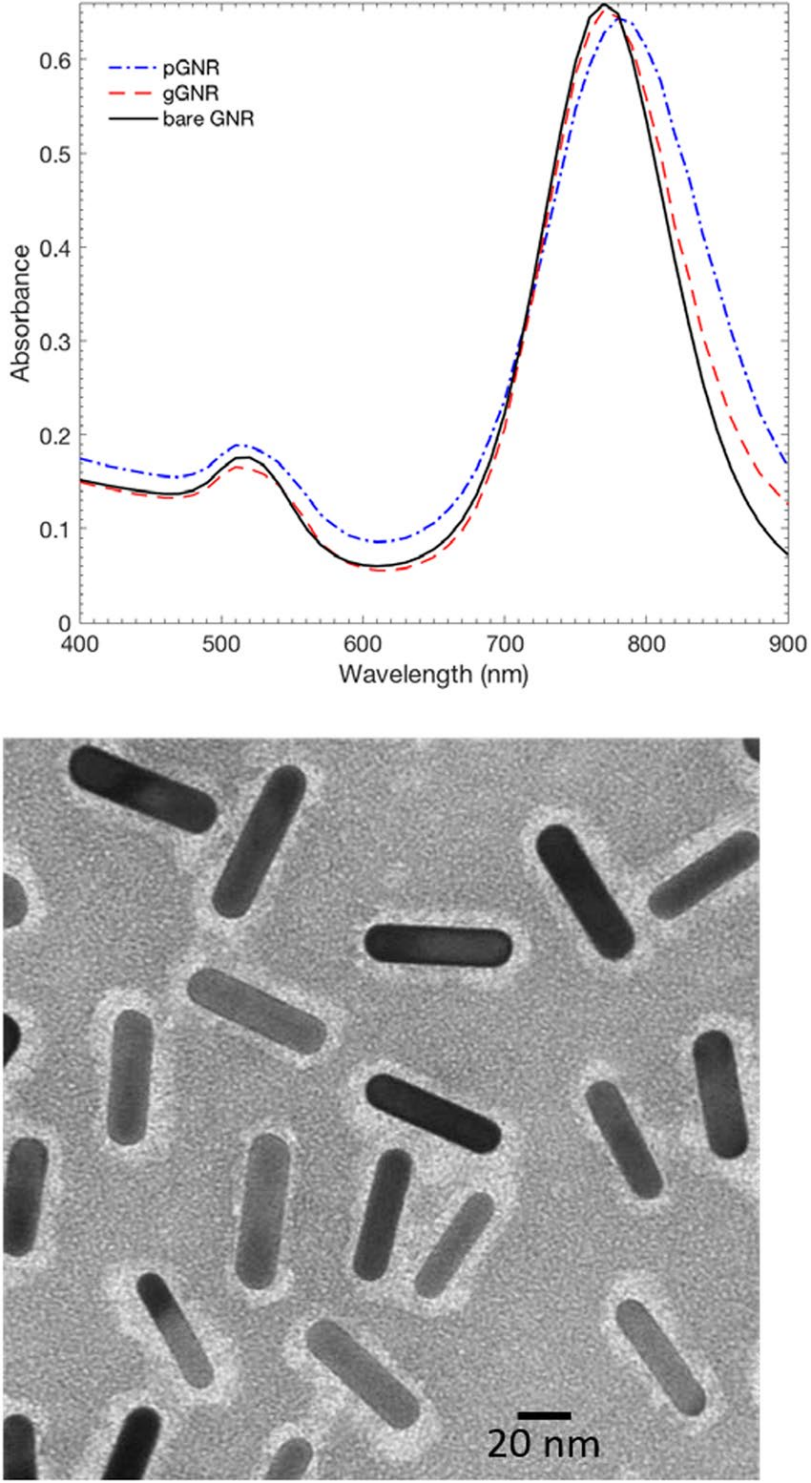
Gold Nanoparticle Characterization. Top: Absorption spectra of bare GNR, pGNR, and gGNR. Spectra for both pGNR and gGNR are consistent with a classical nanorod shape with a short axis absorption near 520 nm and a long axis absorption near 780 nm. Bottom: TEM images of gGNR demonstrate preservation of gold nanorod structure throughout conjugation and a corona that is indicative of the presence of a surface layer of goserelin and PEG.

MicroBCA protein assay using pGNR as a control revealed approximately 1200 molecules of goserelin conjugated to each gGNR. Absorbance spectra for both pGNR and gGNR were consistent with a classical nanorod shape (Fig. 3) with a short axis absorption near 520 nm and a long axis absorption near 780 nm. Compared with pGNR, gGNR had a slight blue shift (8 nm) consistent with successful conjugation of goserelin to PEG. Average zeta potentials (with 95% confidence intervals) for bare GNR, pGNR, and gGNR were + 46 ± 6.3 mV, −4.3 ± 1.8 mV, and – 3.5 ± 0.58 mV, respectively. These values are consistent with efficient removal of positively charged cetyltrimethylammonium bromide (CTAB) from bare GNR.

### *In Vitro* pGNR and gGNR Uptake Studies

**Figure 4:**
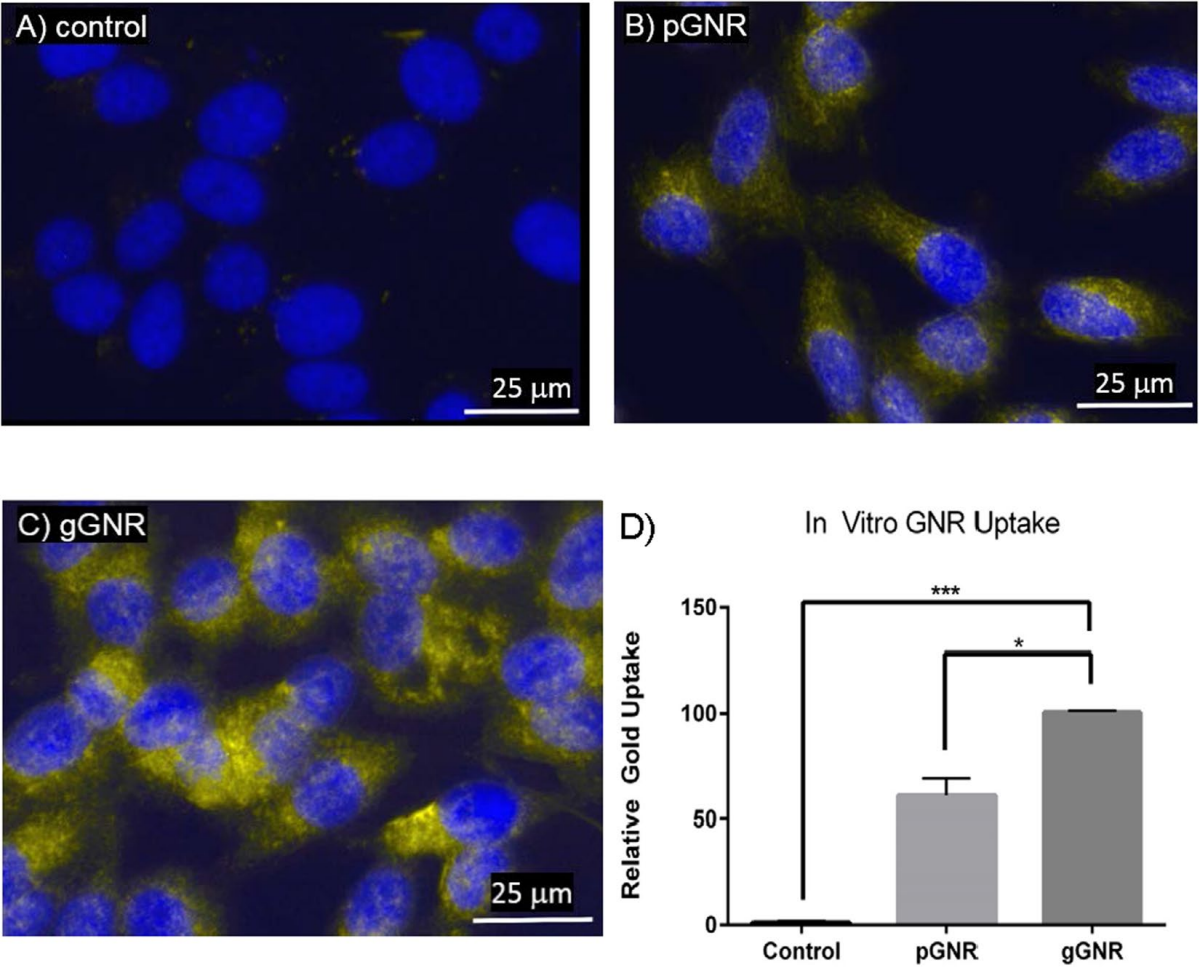
*In vitro* pGNR and gGNR uptake. (**A**–**C**) Darkfield and fluorescence overlay images of PC3 cells incubated with media, pGNR, or gGNR demonstrate qualitatively increased GNR uptake by gGNR compared to pGNR and controls. (**D**) ICP-MS analysis of PC3 cells incubated with media, pGNR, or gGNR demonstrates nearly 1.5 fold increase in gold uptake from gGNR compared to pGNR. Error bars = SEM. *p < 0.05. ***p < 0.001.

Qualitative analysis of GNR uptake using darkfield and fluorescence microscopy demonstrated superior uptake by gGNR compared with pGNR (Fig. 4A–C). Quantitative analysis by ICP-MS demonstrated that gGNR had a nearly 1.5 fold increase in uptake compared to pGNR (average intracellular gold concentration relative to control = 64.6 for pGNR vs. 92.2 for gGNR, p < 0.05) (Fig. 4D). Enhanced uptake by gGNR by PC3 cells was likely due to the high expression of GnRH receptor on the cell surface and efficient binding and internalization of goserelin, the FDA-approved GnRH superagonist. Additionally, darkfield microscopy imaging of normal prostate and PC3 cells treated with gGNRs demonstrated tumor cell-specific uptake of gGNRs (see Supplementary Fig. S1).

### *In Vitro* Clonogenic Assay

**Figure 5:**
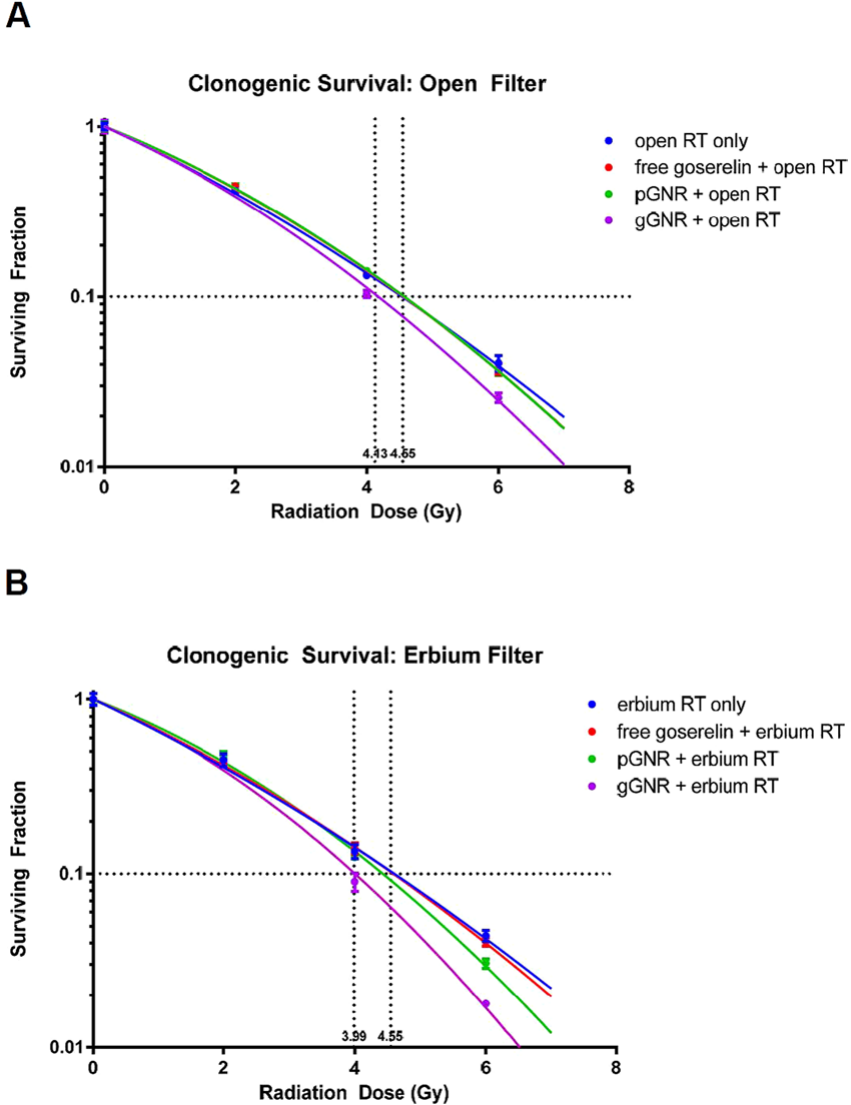
GNR radiosensitization with standard copper-filtered 250 kVp beam with and without erbium filter. Dotted lines intersecting the x axis represent the radiation dose necessary to reduce surviving fraction to 10% for RT only and gGNR + RT conditions. Both clonogenic assays demonstrate that gGNR is a significantly stronger radiosensitizer than pGNR. However, the addition of the erbium filter (**B**) did not provide significantly enhanced gGNR-mediated radiosensitization compared to the standard beam alone (**A**).

Clonogenic assays were performed in parallel experiments using a standard Cu-filtered 250 kVp beam with and without the Er filter. The addition of the Er filter did not provide significantly enhanced gGNR-mediated radiosensitization compared to the standard beam as measured by differences in surviving fraction at 4 Gy (average surviving fraction = 10.3 without erbium filter vs. 9.0 with erbium filter, p = 0.30). Free goserelin controls did not offer any significant degree of radiosensitization, suggesting that gGNR-mediated radiosensitization is due to gold uptake and not the presence of goserelin as demonstrated in previous *in vitro* studies^27^. Dose enhancement factor at 10% surviving fraction (DEF_10%_) was calculated by dividing the dose needed to reduce the surviving fraction to 10% using radiation alone by the dose needed to reach the same endpoint using radiation with gGNR. DEF_10%_ was 1.10% and 1.14% without Er filter (Fig. 5A) and with Er filter (Fig. 5B), respectively, demonstrating that gGNRs are effective radiosensitizers with the Cu-filtered 250 kVp beam regardless of the presence of the Er filter.

### *In Vitro* Gamma H2AX Assay

**Figure 6:**
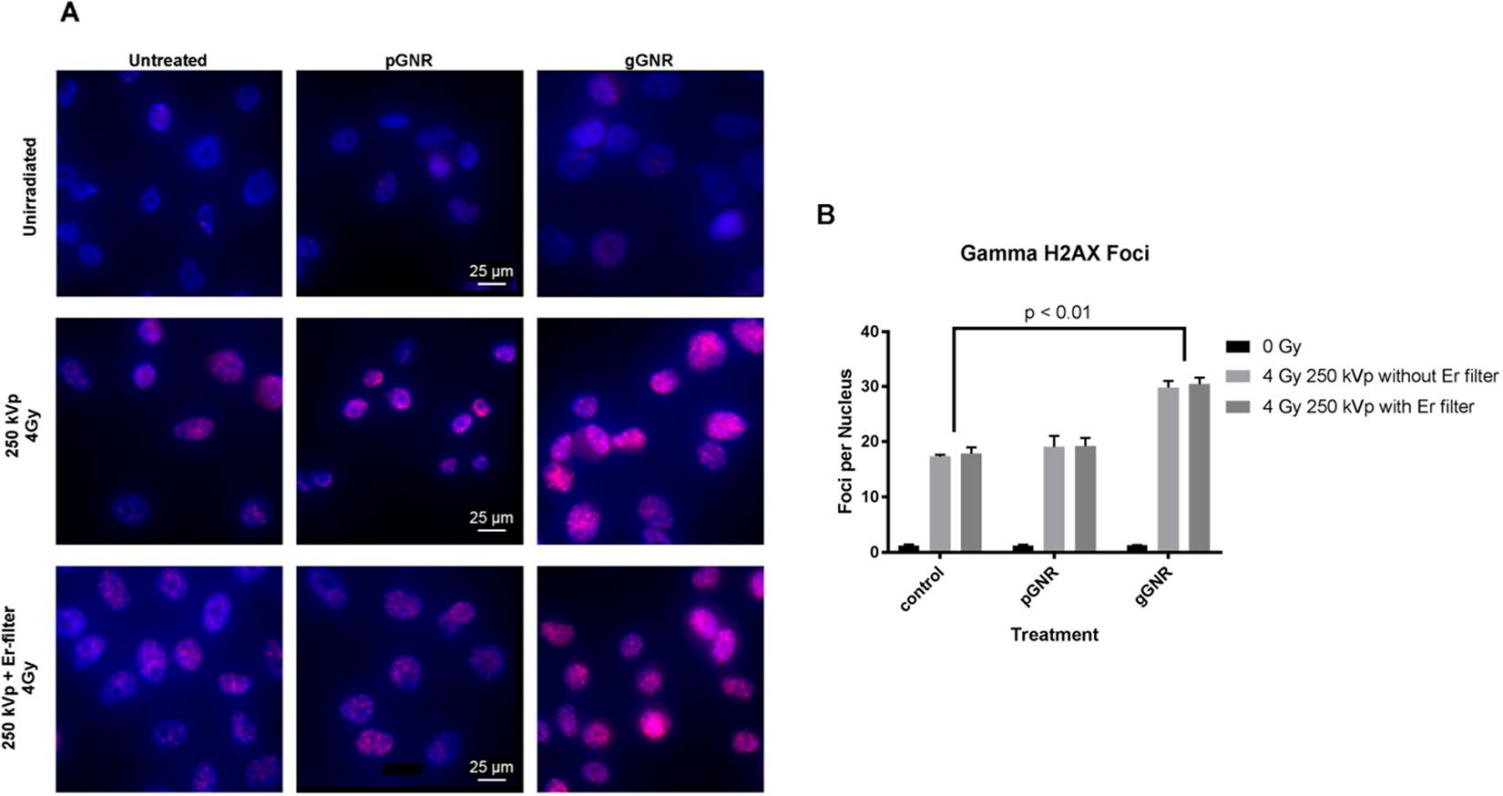
Gamma H2AX Foci Analysis with standard copper-filtered 250 kVp beam with and without erbium filter. (**A**) Fluorescence overlay images showing nuclei (DAPI filter) and gamma H2AX foci (Texas Red filter) demonstrate qualitatively higher foci density with gGNR + RT compared to pGNR + RT or RT only. The scale bars, applicable to all the images, are shown in the middle images only. (**B**) gGNR + RT demonstrated significantly more double stranded DNA breaks compared to pGNR + RT regardless of the presence of the erbium filter. The addition of the erbium filter did not provide significantly enhanced foci density with gGNR + RT compared to the standard beam.

Gamma H2AX assays were performed in parallel experiments using the standard Cu-filtered 250 kVp beam with and without Er filter. gGNR + RT demonstrated significantly more double stranded DNA breaks compared to pGNR + RT regardless of the presence of Er filter (average foci count per cell = 19.1 for pGNR + RT vs. 30.1 for gGNR + RT, p < 0.01) (Fig. 6). The addition of Er filter did not provide significantly enhanced foci density with gGNR + RT compared to the standard beam (average foci count per cell = 29.8 without Er filter vs. 30.4 with Er filter, p = 0.73) (Fig. 6).

### *In Vivo* Tumor Growth Inhibition

**Figure 7:**
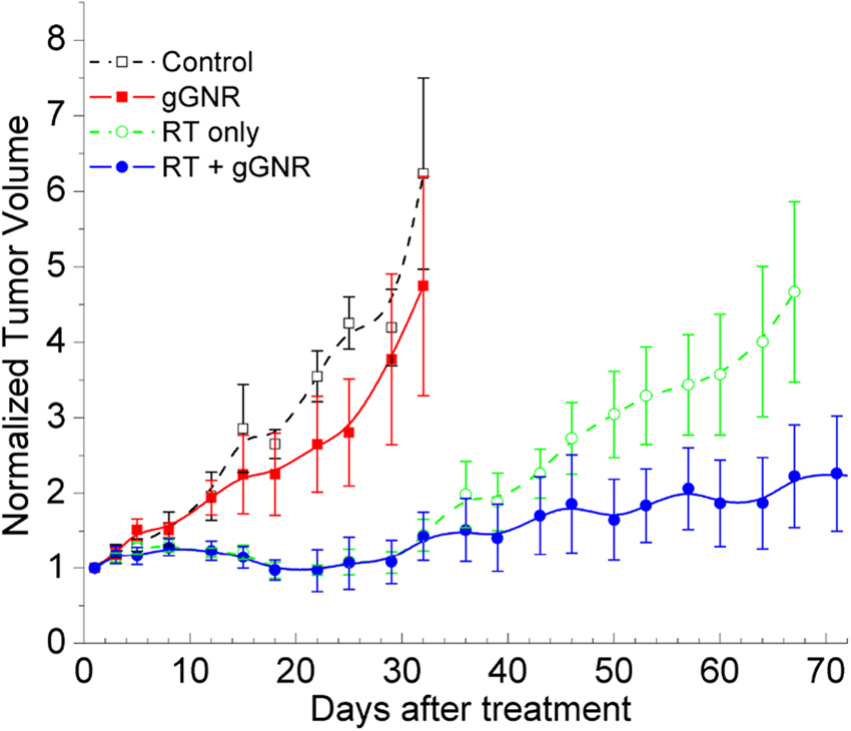
*In vivo* subcutaneous PC3 xenograft tumor growth inhibition. The difference between the two groups (RT + gGNR vs. RT only), in terms of the rate of regrowth for days ≥14, was highly significant (p < 0.0005). By day 64 of the study, RT + gGNR treament of mice resulted in more than a two fold reduction in normalized tumor volume compared with RT alone. Mice treated with gGNR alone had no significant difference in average normalized tumor volume compared with untreated controls. Error bars = SEM.

Adult male nude mice bearing subcutaneous PC3 xenograft tumors were randomized into four groups (untreated control, gGNR alone, RT alone, and RT + gGNR) and underwent tumor volume measurement thrice weekly. RT was performed using a standard Cu-filtered 250 kVp beam with the Er filter at a single 5 Gy dose given 24 hours after intravenous (IV) administration of GNRs. The dose of gGNR was 100 µL of 10 optical density (OD) (42.6 µg gold/mL). Given that the average weight of an experimental mouse is 30 g, the dose of injected gold was about 1.42 µg/g of animal weight. Tumor volumes were nornalized to pretreatment starting volumes and plotted against days after treatment (Fig. 7). The treatment effectiveness was assessed by comparing the slopes of log relative tumor volume vs. days curves. Statistical analysis following this approach suggested that RT + gGNR treatment was considerably more effective than RT only. PC3 xenograft tumors treated with RT + gGNR had a highly significant (p < 0.0005) decrease in the rate of regrowth after day 14 of treatment compared to those treated with RT alone. Mice treated with RT + gGNR had far smaller normalized tumor volume compared with RT alone by day 64 of the study (1.87 vs. 4.01) (Fig. 7). Additionally, mice treated with gGNR alone had no significant difference in average normalized tumor volume compared with untreated controls. This is consistent with our *in vitro* findings that cytotoxicity from GNP-mediated radiosensitization is not due to the direct effects of GNPs or their targeting moieties alone, but rather to the synergistic interaction between radiation and tumor-localized GNPs.

## Discussion

Consistent with previous work using GNPs as radiosensitizers, our results demonstrate that conjugation of GNPs to active targeting moieties results in increased tumor cell uptake and subsequent radiosensitization compared with unconjugated GNP controls^8,28–30^. While unconjugated GNPs rely exclusively on leaky tumor vasculature to reach tumors by the EPR effect, it is clear that tumor-targeted GNPs accumulate to a higher degree within tumor cells, representing an elegant advancement in nanomedicine with promising broad clinical applications. Our results also extend these observations to sensitization of tumors to brachytherapy using a radioisotope (Yb-169) that has an energy spectrum predicted to maximize biological effects of GNP-mediated dose enhancement, paving the way for clinical translation of a tumor-specific brachytherapy strategy.

In our experiments, goserelin served as an excellent targeting moiety taking advantage of GnRH receptor overexpression in prostate cancer cells. It is important to note, however, that the GnRH receptor principally serves as a physiologic receptor in the anterior pituitary, which governs the functions of multiple endocrine organs including the thyroid and adrenal glands. Additionally, GNPs are known to accumulate substantially in the liver and spleen even when conjugated. Thus, future clinical applications of combined goserelin-conjugated GNPs and radiation therapy require that radiation be entirely restricted to solid tumor targets as there is a potential for off-target effects in the liver, spleen, and endocrine organs.

In our experiments, standard Cu-filtered 250 kVp x-rays with and without the Er-filter were equally effective in terms of inducing GNP-mediated radiosensitization of prostate cancer cells as measured by both DNA damage and clonogenic survival. According to the current MC results, the Er-filter shifted the most intense part of a standard Cu-filtered 250 kVp x-ray spectrum to a lower energy range, not only better matching the Yb-169 gamma ray spectrum but also further increasing the theoretical dose enhancement both macroscopically and microscopically because the maximum difference in the mass energy absorption coefficients between gold and water/tissue occurs around the photon energy of ~40 keV. Nevertheless, the current *in vitro* data showed there was no statistically significant difference between the two beams in terms of inducing GNP-mediated radiosensitization. This observation essentially suggests that the currently applied shift (~10 keV) in the locations of XRF peaks in the 250 kVp x-ray spectrum towards the photon energy providing a theoretical maximum of macroscopic dose enhancement exerted insignificant impact on GNP-mediated radiosensitization, at least when measured with classical biological assays for radiosensitization. This was likely because photons needed to interact with sparsely distributed GNPs, although with relatively higher interaction probabilities, resulting in a fairly minor increase in the fluence of secondary electrons. Moreover, since they were produced from photoelectric absorption interactions primarily with gold L-shell electrons, the secondary electrons under the current situation were short-ranged (on the order of 10 s of micrometers or less) and thus further experienced self-absorption within GNPs or clustered GNPs within cellular endosomes. In particular, most Auger/Coster-Kronig electrons (with their ranges on the order of 10 s of nanometers) were likely absorbed within GNPs^12^.

From a biological standpoint, the short-ranged secondary electrons generate free radicals (reactive oxygen species) in the immediate vicinity of the GNP that then travel to the nucleus to cause DNA strand breaks as noted with the gamma H2AX foci. Notably, however, even with receptor-mediated cellular internalization contributing to radiosensitization, free radicals generated by secondary electrons may be absorbed by the endosomal and nuclear membranes prior to reaching their desired DNA target^31^. This compartmentalization of free radicals in mature endosomes that encapsulate the vast majority of intracellular GNPs could limit the potency of radiosensitization^32^. Taken together with physical considerations explained above and limits of sensitivity of biological assays of radiosensitization, these barriers may explain why modest gains in focal dose escalation in the immediate periphery of GNPs achieved by photon energy modulation of 250 kVp beams did not translate into biologically measurable gains in radiosensitization in our experiments. A previous *in vitro* study performed with monochromatic synchrotron x-rays also provided similar inconclusive data for GNP-mediated radiosensitization when the photon beam energy was changed from 60 keV to 50 keV^33^. While this study also reported the maximum *in vitro* GNP-mediated radiosensitization occurring at 40 keV^33^. it is less likely to be generalizable due to many other biological and physical parameters at play for GNP-mediated radiosensitization *in vitro* and *in vivo*. Overall, the current results illustrate the difficulty in accurately predicting the degree of GNP-mediated radiosensitization, purely based on physical considerations and simplistic computational models (of cells and GNP distributions).

Overall, the current *in vitro* and *in vivo* results have considerable significance, especially in terms of the two important aspects described below. First, they strongly suggest clinical significance of brachytherapy implementation of GNP-mediated dose enhancement/radiosensitization strategy, in conjunction with gGNRs and an Yb-169 source. While not fully emulating the Yb-169 gamma ray spectrum, x-ray spectra of the current external beam surrogates of Yb-169 source were found effective in terms of inducing clinically significant levels of GNP-mediated radiosensitization both *in vitro* and *in vivo* as much as up to ~14 and ~100%, respectively. Due to the limited scope of the current proof-of-principle study, no further attempt was made to identify the exact mechanisms of the observed difference between *in vitro* and *in vivo* results. However, we note several potentially different consequences from our *in vitro* and *in vivo* experiments. Most notably, as with previous studies, there was a good likelihood that irradiation of xenograft tumors treated with targeted GNRs caused tumor vascular damage triggering some detrimental downstream effects resulting in increased shrinkage of tumors or hindering further tumor growth^8,34,35^. This is likely due to a decreasing gradient of nanoparticles away from the tumor vasculature towards the tumor core. The geometric/structural differences between our *in vitro* cells and *in vivo* tumors resulting in different patterns of local dose enhancement may also explain the discrepancy in our results. We anticipate future investigations focusing on these aspects will help elucidate the mechanisms responsible for the currently observed difference between *in vitro* and *in vivo* results. Especially, we envision future *in vitro* and *in vivo* studies will become even more meaningful, if performed with an actual high dose rate Yb-169 source as well as the same external beam surrogates as used in the current study. Our preliminary physical measurements using novel low dose rate Yb-169 sources and GNP-loaded radiochromic dosimeters suggested that the Yb-169 gamma ray spectrum could be considerably more effective (>50%) than x-ray spectra of the current external beam surrogates of Yb-169, in terms of creating free radicals due to the presence of GNPs in tissue-like media^36^. Although it needs to be confirmed by a more comprehensive investigation, this result suggests that the Yb-169 spectral lines above the gold *K-*absorption edge could play a more important role for GNP-mediated dose enhancement and radiosensitization than originally expected.

The second important aspect of the current results is that clinically significant radiosensitization was achievable with just intravenous injection of trace amounts of GNPs (on the order of 1 µg/g of animal weight) although no significant GNP-mediated dose enhancement was expected to occur based on macroscopic estimation^37^. While it is puzzling at a glance, this seeming contradiction can be explained at least qualitatively based on the concept known as microscopic dose enhancement that we originally proposed and demonstrated in our MC study^23^. According to our original and subsequent MC studies^23,24^, microscopic dose enhancement (up to a factor of 100 or more) occurs in the immediate vicinity (<~10 µm) of GNPs irradiated by kilovoltage x-rays (or keV-range gamma rays such as those emitted from Yb-169). Thus, if the intracellular or intratumoral locations of GNPs were close enough to cellular organelles critical for cell function and survival (e.g., nucleus, mitochondria, etc.), the secondary electrons emitted from GNPs could still result in irreversible damage (e.g., DNA double strand breaks) to tumor cells as well as endothelial cells lining the tumor vasculature, either directly or indirectly (via free radical production along the secondary electron tracks). In fact, other researchers applied a similar concept, in conjunction with an approach often known as a local effect model^38,39^, to develop biological outcome models for GNP-mediated radiosensitization and were able to produce quantitative results^12,40^ that reasonably matched *in vitro* experimental results under the situations where macroscopic estimation of GNP-mediated dose enhancement becomes less relevant. For example, these outcome models could be used to explain some of the puzzling observations made from *in vitro* studies including ours such as significant GNP-mediated radiosensitization with 6 MV x-rays and/or trace amounts of GNPs^8,19,41,42^. Nevertheless, their reliance on considerable empiricism has prompted the necessity to develop a predictive model with minimal or no empiricism. At this time, the quest for such a predictive model appears to be challenging, primarily due to the fact that exact biological and physicochemical mechanisms for GNP-mediated radiosensitization and their interplay are not well known, although it has been shown that, besides GNP-mediated dose enhancement, the key mechanisms at play include GNP-induced oxidative stress, cell cycle disruption, and DNA repair inhibition^20,29,43,44^. Moreover, there is considerable variation within the literature regarding GNP-mediated radiosensitization due to the wide range of GNP formulations, surface chemistry, and cell types/tumor models employed^12,45^. Therefore, coordinated research efforts using standardized computational and experimental procedures will be necessary to drastically improve our understanding of GNP-mediated dose enhancement and radiosensitization so that this novel and elegant radiosensitization strategy will ultimately be ready for clinical translation in the future.

## Methods

### Modulation of the 250 kVp X-ray Spectrum Using an Erbium Foil

While it was not technically feasible to perfectly match discrete gamma ray spectrum by modulating continuous bremsstrahlung x-ray spectrum, we noted the gamma ray spectrum of Yb-169 has an intensity-weighted average energy of 92.8 keV and about two-thirds of the photons between ~49 and ~63 keV. Thus, we focused on modulating the spectrum of a typical 250 kVp x-ray beam (filtered by 0.35 mm copper; average energy of 89.3 keV) available from a commercial orthovoltage x-ray unit (Philips RT-250) using an exotic metal filter made of a thin (0.25 mm) erbium foil. Specifically, while the Cu-filtered 250 kVp spectrum closely matches the Yb-169 gamma ray spectrum in terms of the average energy, the most intense part of the spectrum occurs around the tungsten K-shell fluorescence x-ray peaks at 59.3 keV (*k*_*α*_) and 67.2 keV (*k*_*β*_), a higher energy range than that (50–63 keV) for the Yb-169 gamma-ray spectrum. To further improve on this aspect, we adopted an approach, similar to what is often known as K-edge filtering^46–48^, in which further filtration of the Cu-filtered 250 kVp x-ray spectrum using an erbium filter resulted in replacing K-shell fluorescence x-rays from the tungsten target with those from the erbium filter, 49.1 keV (*k*_*α*_) and 55.7 keV (*k*_*β*_). The entire task was performed using our experimentally validated MC model of Philips RT-250 x-ray unit and x-ray spectrum measurements as described in detail elsewhere^49,50^.

### Monte Carlo Calculations of the Secondary Electron Spectra within a GNP-loaded Tumor

GNP-mediated dose enhancement, especially on a nano- or cellular-scale, is closely related with the spectrum of secondary electrons emitted from GNPs^23^. Therefore, the first-hand assessment of the dose enhancing capability of a given filtered x-ray spectrum can be achieved by calculating such a secondary electron spectrum. In this investigation, by applying our own MC techniques developed previously^23,24^, we determined the secondary electron spectra within a tumor loaded with GNPs at 7 mg/g for various filtered 250 kVp x-ray beams as well as a hypothetical Yb-169 beam (conceptually similar to other isotope-based beams such as a Co-60 beam). Briefly, the MC geometry model consisted of a 1 × 1 × 1 cm^3^ tumor sitting on top of a 30 × 30 × 29 cm^3^ tissue phantom irradiated by each photon beam at 50 cm source-to-surface distance (SSD). The EGSnrc^51^ and user code DOSXYZnrc^52^ were modified to allow the scoring of the secondary electrons including Auger/Coster-Kronig electrons with their energy down to 25 eV during MC simulations. Four billion histories were followed for each simulation. As in the previous studies^6,23,53^, four-component ICRU tissue (i.e. 10.1% hydrogen, 11.1% carbon, 2.6% nitrogen and 76.2% oxygen) was used as the phantom material and a uniform mixture between gold and four-component ICRU tissue was used to approximate a GNP-loaded tumor, which was deemed acceptable within the scope of this particular task.

### Gold Nanoparticle Synthesis and Characterization

**Figure 8:**
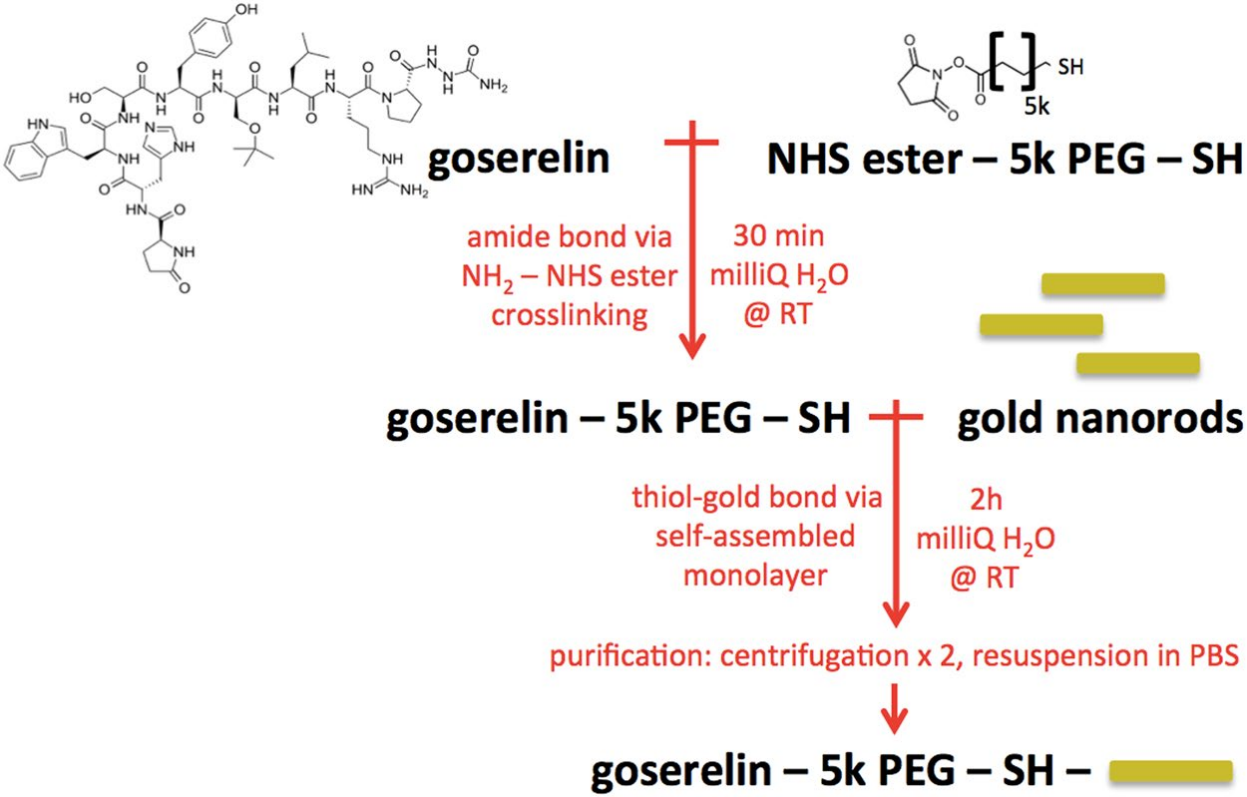
Two step gGNR conjugation. First, goserelin is covalently bonded to thiolated 5k PEG via amine and NHS ester crosslinking chemistry. Then, this complex is conjugated to gold nanorods via thiol-gold bonds. The final construct is then purified from unreacted complexes by two rounds of centrifugation.

Bare GNRs stabilized in 1 mM CTAB with a longitudinal surface plasmon resonance peak of 780 nm were purchased from a commercial vendor (NanoHybrids). OD was measured at 780 nm and used to quantify the concentration of gold. Peptide conjugation to goserelin was based on the method developed by Wolfe *et al*.^8^ (Fig. 8). Briefly, 0.5 mL of 4 mM thiol-PEG-NHS ester (NanoCS PG2-NSTH-5k) was added to 2 mL of 3.2 mM goserelin acetate (Toronto Research Chemicals) and reacted at room temperature for 30 minutes in milliQ water. Then, 2 mL of 10 OD GNRs diluted in milliQ water was added to the reaction and allowed to pegylate at room temperature for 2 hours. The resulting goserelin conjugated GNRs (gGNRs) were separated from unbound reactants via centrifugation at 6,500 g for 30 minutes. Purified gGNRs were then resuspended in phosphate-buffered saline (PBS) (HyClone SH30256) and centrifuged again to ensure complete removal of unbound goserelin. As a comparable control, pegylated GNRs (pGNRs) were made by reacting 0.5 mL of 4 mM 5k methoxy-PEG-thiol (NanoCS PG1-TH-5k) with 2 mL of 10 OD GNRs diluted in milliQ water at room temperature for 2 hours followed by the same purification and wash steps outlined above. To ensure successful conjugation of goserelin to gGNR, samples of gGNR and pGNR with identical 562 nm absorbance in a UV-clear 96 well plate (Corning 3635) was reacted with bicinchoninic acid (ThermoFisher MicroBCA Protein Assay Kit 23235) for two hours at 37 °C, then read at 562 nm using a UV spectrophotometer (BioTek Cytation 5). The resulting difference in 562 nm absorbance was assumed to arise from goserelin protein present in gGNR and used to calculate the number of goserelin molecules conjugated to each GNR. pGNR and gGNR were further characterized by absorbance spectra (BioTek Cytation5 with 1 cm path length) and zeta potential (Malvern ZetaSizer Nano ZS).

### pGNR and gGNR Uptake Studies

To *quantitatively* measure cellular internalization of pGNR and gGNR, PC3 cells grown in 60 mm petri dishes (Corning 353004) were incubated with 2 mL of complete media, 0.5 OD pGNR, or 0.5 OD gGNR for 24 hours. All nanoparticles were diluted to their final concentration in complete media from 50x stock preparations. After washing twice with PBS, cells were trypsinized with 0.5 mL of 0.05% trypsin-EDTA (ThermoFisher 25300-054), neutralized with 2 mL of complete media, and centrifuged at 400 g for 5 minutes. Cells were then resuspended in 1 mL of PBS, transferred to glass scintillation vials (Sigma-Aldrich Z190527), and evaporated at 56 °C overnight. Cells were digested in aqua regia made from OPTIMA grade hydrochloric acid (Fisher A466-1) and nitric acid (Fisher A467-1) for 2 hours and diluted with 4 mL of 1% hydrochloric acid. Gold in cell samples was quantified by ICP-MS against serially diluted elemental gold standards (Agilent 5190-8282) and normalized to cell count. To *qualitatively* measure cellular internalization of pGNR and gGNR, cells were grown in 8 well chamber slides (ThermoFisher 154453) and incubated with media, 2 OD pGNR, or 2 OD gGNR for 24 hours before being stained with 2 µg/mL Hoechst 33342 and prepared for visualization using ProLong® Gold Antifade Mountant (ThermoFisher P10144). Chamber slides were visualized by fluorescence and darkfield microscopy (Leica DM1000). In addition, RWPE-1 cells cultured in keratinocyte serum free medium (Invitrogen) were treated similarly with 2 OD pGNR or 2 OD gGNR for 24 hours and visualized by darkfield microscopy (CytoViva) to compare the uptake in normal cells to that in prostate cancer cells.

### *In Vitro* Clonogenic Assay

To assess *in vitro* radiosensitization, PC3 cells grown in 60 mm petri dishes were incubated with 2 mL of complete media, free goserelin, 0.05 OD pGNR, or 0.5 OD gGNR. Free goserelin was diluted in complete media at a concentration of 3 µg/mL, the same goserelin concentration found in 0.5 OD gGNR as derived by micro BCA assay outlined above. Once again, nanoparticles were diluted to their final concentration from 50x stock preparations. After 24 hours, cells were washed twice with PBS and exposed to Cu-filtered 250 kVp x-rays (Philips RT-250) at 0, 2, 4, or 6 Gy with or without the Er filter. The dose rates (in water) for the Cu-filtered 250 kVp x-ray beam with and without the Er filter were determined by ion chamber measurements following the AAPM TG-61 protocol^54^ and found to be 48 cGy/min and 115 cGy/min under the standard condition (15 mA, 10 cm × 10 cm field size, and 50 cm source-to-chamber/object distance), respectively. Immediately following x-ray irradiation, cells were counted and reseeded in 6 well plates (Corning 3516) and incubated for 10 days before being stained with crystal violet. Plating efficiency was calculated by the number of colonies present in non-irradiated wells divided by the number of cells seeded. Surviving fraction for each treatment condition was calculated by dividing the number of colonies by the product of the number of cells seeded times the plating efficiency. Dose enhancement factor at 10% surviving fraction (DEF_10%_) was calculated by dividing the radiation dose needed to reduce the surviving fraction to 10% in the radiation only group by the dose needed to reach the same endpoint in the radiation + GNR groups. In total, two independent clonogenic experiments were performed, each with six replicates.

### *In Vitro* Gamma H2AX Assay

To assess DNA double stranded breaks due to GNR-mediated radiosensitization, PC3 cells were grown in 8-well chamber slides and incubated for 24 hours with 0.2 mL of complete media, 0.5 OD pGNR, or 0.5 OD gGNR. Nanoparticles were diluted to their final concentration in complete medial from 50x stock preparations. Cells were then washed twice with PBS and exposed to Cu-filtered 250 kVp x-rays (Philips RT-250) at 0 or 4 Gy with or without the Er filter. One hour after irradiation, cells were washed and fixed with 2% formaldehyde for 10 minutes. Subsequently, cells were permeabilized with 0.5% Triton-100x for 10 minutes, blocked with 5% fetal bovine serum (FBS), then stained overnight at 4 °C with Alexa Fluor® 488 conjugated anti-gamma H2A.X-phosphorylated (ser139) antibody (BioLegend 613410) at a concentration of 5 µg/mL in 5% FBS. Then, chamber slides were washed twice with PBS, counterstained with 2 µg/mL Hoechst 33342, and prepared for visualization using ProLong® Gold Antifade Mountant (P10144). Chamber slides were visualized by fluorescence imaging using DAPI and Texas Red filters (BioTek Cytation 5). Foci were scored manually by the same user and averaged over at least 50 nuclei for each treatment condition.

### Animals and Tumor Inoculation

The animals used in this study were male Swiss nude mouse bearing subcutaneous human prostate cancer xenografts induced via injection of 0.05 mL of 2 × 10^6^ PC3 cells (PC3 cell line; American Type Culture Collection, Manassas, VA) in PBS into the right lateral thigh. At the time of this investigation, the mouse was 8 weeks old, weighed 30 g, and the tumor had reached approximately 7 mm in diameter. All experimental animal studies were carried out in accordance with protocols and guidelines approved by the Institutional Animal Care and Use Committee (IACUC) of The University of Texas MD Anderson Cancer Center.

### GNR Injection and Irradiation in Animals

Tumor-bearing mice were randomized by starting volume into four groups containing 6–7 animals each: control, gGNR only, RT only, and RT + gGNR. The animals in the gGNR groups received 100 µL of 10 OD gGNR intravenously. Animals in the RT groups received a single 5 Gy dose of standard Cu-filtered 250 kVp beams with the Er filter using a Philips RT-250 orthovoltage unit equipped with a cone applicator to restrict radiation toward the tumor target. Radiation was administered 24 h after gGNR administration. For animal irradiation, an experimentally measured cone application factor was applied to properly adjust the dose rate determined under the standard condition. Animals were anesthetized using a ketamine and xylazine mixture delivered by intraperitoneal injection prior to irradiation.

### *In Vivo* Tumor Growth Inhibition

Tumors were measured in cubic millimeters using calipers by the same operator along their long axis (L) and short axis (S) one day prior to treatment initiation for their starting volumes, then 2–3 times per week post-treatment. Tumor volume was calculated using the equation L × S^2^/2. Post-treatment tumor volumes were normalized to the starting tumor volume for each animal.

### Statistical Analysis

For *in vitro* and *in vivo* experimental data, the mean values and associated standard errors of the mean (SEM) values were calculated. The Student’s t-test was utilized to assess the difference between the groups during *in vitro* experiments. A two-tailed p value of < 0.05 was considered as statistically significant. The treatment effectiveness between the two main groups (RT only vs. RT + gGNR) during *in vivo* experiment was assessed by comparing the slope of the regrowth curve after plotting the mean of log relative volume vs. day after the treatment for each group. This analysis was performed using STATA 14 software (STATA Corp., College Station, TX).

## Electronic supplementary material

Supplementary Information

## References

[1] Lawrence TS, Eisbruch A, Shewach DS (1997). Gemcitabine-mediated radiosensitization. Semin Oncol.

[2] Greenberger JS, Epperly MW (2007). Review. Antioxidant gene therapeutic approaches to normal tissue radioprotection and tumor radiosensitization. In Vivo.

[3] Kunz-Schughart LA (2017). Nanoparticles for radiooncology: Mission, vision, challenges. Biomaterials.

[4] Unezaki S (1996). Direct measurement of the extravasation of poleyethyleneglycol-coated liposomes into solid tumor tissue by *in vivo* fluorescence microscopy. International Journal of Pharmaceutics.

[5] Qian X (2008). *In vivo* tumor targeting and spectroscopic detection with surface-enhanced Raman nanoparticle tags. Nat. Biotechnol..

[6] Cho SH (2005). Estimation of tumour dose enhancement due to gold nanoparticles during typical radiation treatments: a preliminary Monte Carlo study. Phys. Med. Biol..

[7] Hainfeld JF, Slatkin DN, Smilowitz HM (2004). The use of gold nanoparticles to enhance radiotherapy in mice. Phys. Med. Biol..

[8] Wolfe T (2015). Targeted gold nanoparticles enhance sensitization of prostate tumors to megavoltage radiation therapy *in vivo*. Nanomedicine.

[9] Chang MY (2008). Increased apoptotic potential and dose-enhancing effect of gold nanoparticles in combination with single-dose clinical electron beams on tumor-bearing mice. Cancer Sci.

[10] Hainfeld JF, Smilowitz HM, O’Connor MJ, Dilmanian FA, Slatkin DN (2013). Gold nanoparticle imaging and radiotherapy of brain tumors in mice. Nanomedicine.

[11] Hainfeld JF (2010). Gold nanoparticles enhance the radiation therapy of a murine squamous cell carcinoma. Physics in Medicine and Biology.

[12] Schuemann J (2016). Roadmap to Clinical Use of Gold Nanoparticles for Radiation Sensitization. Int. J. Radiat. Oncol. Biol. Phys..

[13] Bhattarai SR (2017). Gold nanotriangles: scale up and X-ray radiosensitization effects in mice. Nanoscale.

[14] Chauhan VP (2011). Fluorescent nanorods and nanospheres for real-time *in vivo* probing of nanoparticle shape-dependent tumor penetration. Angew Chem Int Ed Engl.

[15] Xiao F (2011). On the role of low-energy electrons in the radiosensitization of DNA by gold nanoparticles. Nanotechnology.

[16] Carter JD, Cheng NN, Qu Y, Suarez GD, Guo T (2007). Nanoscale energy deposition by X-ray absorbing nanostructures. J Phys Chem B.

[17] Cho SH, Jones BL, Krishnan S (2009). The dosimetric feasibility of gold nanoparticle-aided radiation therapy (GNRT) via brachytherapy using low-energy gamma-/x-ray sources. Phys. Med. Biol..

[18] Montenegro M, Nahar SN, Pradhan AK, Huang K, Yu Y (2009). Monte Carlo Simulations and Atomic Calculations for Auger Processes in Biomedical Nanotheranostics. Journal of Physical Chemistry A.

[19] Chithrani DB (2010). Gold nanoparticles as radiation sensitizers in cancer therapy. Radiat. Res..

[20] Butterworth KT, McMahon SJ, Currell FJ, Prise KM (2012). Physical basis and biological mechanisms of gold nanoparticle radiosensitization. Nanoscale.

[21] Roeske JC, Nunez L, Hoggarth M, Labay E, Weichselbaum RR (2007). Characterization of the theorectical radiation dose enhancement from nanoparticles. Technol. Cancer Res. Treat..

[22] Lechtman E (2011). Implications on clinical scenario of gold nanoparticle radiosensitization in regards to photon energy, nanoparticle size, concentration and location. Phys. Med. Biol..

[23] Jones BL, Krishnan S, Cho SH (2010). Estimation of microscopic dose enhancement factor around gold nanoparticles by Monte Carlo calculations. Med. Phys..

[24] Reynoso FJ, Manohar N, Krishnan S, Cho SH (2014). Design of an Yb-169 source optimized for gold nanoparticle-aided radiation therapy. Med. Phys..

[25] Ngwa W (2013). *In vitro* radiosensitization by gold nanoparticles during continuous low-dose-rate gamma irradiation with I-125 brachytherapy seeds. Nanomedicine.

[26] Moeendarbari S (2016). Theranostic Nanoseeds for Efficacious Internal Radiation Therapy of Unresectable Solid Tumors. Sci Rep.

[27] Hermann RM (2007). No supra-additive effects of goserelin and radiotherapy on clonogenic survival of prostate carcinoma cells *in vitro*. Radiat Oncol.

[28] Gargioni E (2016). Targeted nanoparticles for tumour radiotherapy enhancement-the long dawn of a golden era?. Ann Transl Med.

[29] Kang B, Mackey MA, El-Sayed MA (2010). Nuclear targeting of gold nanoparticles in cancer cells induces DNA damage, causing cytokinesis arrest and apoptosis. J Am Chem Soc.

[30] Cai ZL (2017). Local Radiation Treatment of HER2-Positive Breast Cancer Using Trastuzumab-Modified Gold Nanoparticles Labeled with Lu-177. Pharm. Res..

[31] Fisher AB (2009). Redox signaling across cell membranes. Antioxid Redox Signal.

[32] Ganley IG, Carroll K, Bittova L, Pfeffer S (2004). Rab9 GTPase regulates late endosome size and requires effector interaction for its stability. Mol Biol Cell.

[33] Rahman WN (2014). Optimal energy for cell radiosensitivity enhancement by gold nanoparticles using synchrotron-based monoenergetic photon beams. Int J Nanomedicine.

[34] Chattopadhyay N (2013). Molecularly targeted gold nanoparticles enhance the radiation response of breast cancer cells and tumor xenografts to X-radiation. Breast Cancer Res Treat.

[35] Yang Y (2016). Tumor Angiogenesis Targeted Radiosensitization Therapy Using Gold Nanoprobes Guided by MRI/SPECT Imaging. ACS Appl Mater Interfaces.

[36] Cho J, Alqathami M, Reynoso F (2016). & Cho, S. TU-H-CAMPUS-TeP3-04: Probing the Dose Enhancement Due to a Clinically-Relevant Concentration of Gold Nanoparticles and Yb-169 Gamma Rays Using PRESAGE Dosimeters. Med Phys.

[37] Cho, S. H. & Jones, B. L. in *Cancer Nanotechnology: Principles and Applications in Radiation Oncology Imaging in Medical* Diagnosis *and Therapy* (eds S H Cho & S Krishnan) Ch. 10, 123–136 (Taylor & Francis, 2013).

[38] Elsasser T, Kramer M, Scholz M (2008). Accuracy of the local effect model for the prediction of biologic effects of carbon ion beams *in vitro* and *in vivo*. Int. J. Radiat. Oncol. Biol. Phys..

[39] Elsasser T, Scholz M (2007). Cluster effects within the local effect model. Radiat. Res..

[40] McMahon SJ (2011). Biological consequences of nanoscale energy deposition near irradiated heavy atom nanoparticles. Scientific Reports.

[41] Jain S (2011). Cell-Specific Radiosensitization by Gold Nanoparticles at Megavoltage Radiation Energies. International Journal of Radiation Oncology Biology Physics.

[42] Berbeco RI (2012). DNA Damage Enhancement from Gold Nanoparticles for Clinical MV Photon Beams. Radiat. Res..

[43] Taggart LE, McMahon SJ, Currell FJ, Prise KM, Butterworth KT (2014). The role of mitochondrial function in gold nanoparticle mediated radiosensitisation. Cancer Nanotechnol.

[44] Cui L (2014). Hypoxia and cellular localization influence the radiosensitizing effect of gold nanoparticles (AuNPs) in breast cancer cells. Radiat Res.

[45] Her S, Jaffray DA, Allen C (2015). Gold nanoparticles for applications in cancer radiotherapy: Mechanisms and recent advancements. Adv Drug Deliv Rev.

[46] Beaman S, Lillicrap SC, Price JL (1983). Tungsten anode tubes with K-edge filters for mammography. Br. J. Radiol..

[47] McDonagh CP, Leake JL, Beaman SA (1984). Optimum x-ray spectra for mammography: choice of K-edge filters for tungsten anode tubes. Phys. Med. Biol..

[48] Jennings RJ, Eastgate RJ, Siedband MP, Ergun DL (1981). Optimal x-ray spectra for screen-film mammography. Med. Phys..

[49] Reynoso FJ, Tailor R, Wang CKC, Cho SH (2016). Comparison of filtered x-ray spectra and depth doses derived from a hybrid Monte Carlo model of an orthovoltage x-ray unit with experimental measurements. Biomedical Physics & Engineering Express.

[50] Reynoso, F. J. *Design of an ytterbium-169 brachytherapy source for gold nanoparticle-aided radiation therapy* PhD thesis, Georgia Institute of Technology, (2014).

[51] Kawrakow, I. & Rogers, D. The EGSnrc code system: Monte Carlo simulation of electron and photon transport. *NRC Report PIRS* **701** (2000).

[52] Walters, B., Kawrakow, I. & Rogers, D. DOSXYZnrc users manual. *NRC Report PIRS* **794** (2005).

[53] Reynoso FJ, Munro Iii JJ, Cho SH (2017). Technical Note: Monte Carlo calculations of the AAPM TG-43 brachytherapy dosimetry parameters for a new titanium-encapsulated Yb-169 source. J. Appl. Clin. Med. Phys..

[54] Ma C-M (2001). AAPM protocol for 40–300 kV x-ray beam dosimetry in radiotherapy and radiobiology. Med. Phys..

