# Supplementary material for: Radiosensitization of Prostate Cancers In Vitro and In Vivo to Erbium-filtered Orthovoltage X-rays Using Actively Targeted Gold Nanoparticles
Source: Sci Rep. 2017 Dec 22;7:18044. doi: 10.1038/s41598-017-18304-y (PMC5741750; doi:10.1038/s41598-017-18304-y)
Supplement: Supplementary file 1 — Supplementary Information [file 41598_2017_18304_MOESM1_ESM.pdf]

Supplementary Information

Radiosensitization of Prostate Cancers *In Vitro* and *In Vivo* to Erbium-filtered Orthovoltage X-rays Using Actively Targeted Gold Nanoparticles

Allison M. Khoo, Sang Hyun Cho, Francisco J. Reynoso, Maureen Aliru, Kathryn Aziz, Monica Bodd, Xi Yang, Md F. Ahmed, Selcuk Yasar, Nivedh Manohar, Jongmin Cho, Ramesh Tailor, Howard D. Thames, Sunil Krishnan

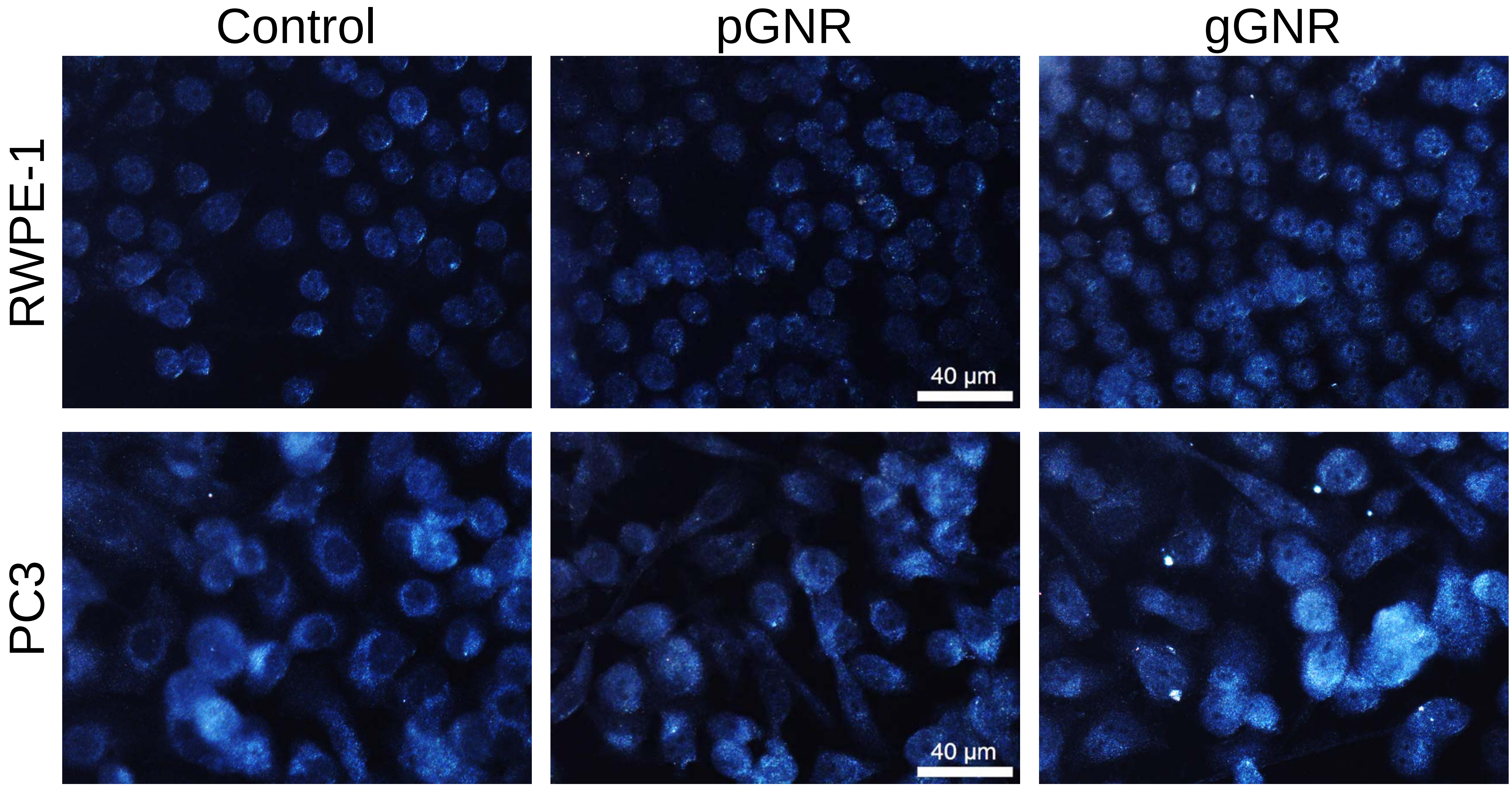

Fig. S1. **Selective uptake of gGNRs by prostate cancer cells.** Dark field microscopy images of PC3 cells and RWPE-1 normal prostate cells incubated with media, pGNR, or gGNR demonstrate qualitatively increased GNR uptake by gGNR compared to pGNR and controls in PC3 cells but no difference in RWPE-1 cells. The scale bars, applicable to all the images, are shown in the middle images only.
